# The impacts of performance-based assessment on reading comprehension achievement, academic motivation, foreign language anxiety, and students’ self-efficacy

**DOI:** 10.1186/s40468-022-00202-4

**Published:** 2022-11-12

**Authors:** Tahereh Heydarnejad, Fariba Tagavipour, Indrajit Patra, Ayman Farid Khafaga

**Affiliations:** 1 Department of English Language, Faculty of Literature and Humanities, University of Gonabad, Gonabad, Iran; 2 English Language Department, Dezful Branch, Islamic Azad University, Tehran, Iran; 3 NIT Durgapur, Durgapur, West Bengal India; 4 Department of English, College of Science and Humanities, Prince Sattam Bin Abdulaziz University, Al-Kharj, Saudi Arabia; 5 Department of English, Faculty of Arts and Humanities, Suez Canal University, Ismailia, Egypt

**Keywords:** Performance-based assessment, Reading comprehension achievement, Academic motivation, Foreign language anxiety, Students’ self-efficacy, EFL learners

## Abstract

The types of assessment tasks affect the learners’ psychological well-being and the process of learning. For years, educationalists were in search of finding and implementing accurate and convenient approaches to assess learners efficiently. Despite the significant role of performance-based assessment (PBA) in affecting second/foreign language (L2) learning processes, few empirical studies have tried to explore how PBA affects reading comprehension achievement (RCA), academic motivation (AM), foreign language anxiety (FLA), and students’ self-efficacy (SS-E). To fill this lacuna of research, the current study intended to gauge the impact of PBA on the improvement of RCA, AM, FLA, and SS-E in English as a foreign language (EFL) context. In so doing, a sample of 88 intermediate EFL learners were randomly divided into experimental group (EG) and CG (control group). During this research (16 sessions), the learners in the CG (*N* = 43) received the tradition assessment. In contrast, the learners in the EG (*N* = 45) were exposed to some modification based on the underpinning theories of PBA. Data inspection applying the one-way multivariate analysis of variance (i.e., the one-way MANOVA) indicated that the learners in the EG outperformed their counterparts in the CG. The results highlighted the significant contributions of PBA in fostering RCA, AM, FLA, and S-E beliefs. The implications of this study may redound to the benefits of language learners, teachers, curriculum designers, and policy makers in providing opportunities for further practice of PBA.

## Introduction

In educational settings, teaching and assessment are the two sides of the same coin. The way teachers teach implicitly and explicitly affects the assessment. That is, the mode of assessment is associated with the teaching approaches and the employed procedures. Such close relationships call for educational and psychological research to consider practical approaches in the development of curriculum, instruction, and assessment in all fields of study, and EFL context is no exception. In the domain of assessment, the traditional assessment was used for years, which encountered different drawbacks. As a reaction to various deficiencies of the traditional assessment, alternatives were proposed (Xu et al, 2022; Yan, 2021; Yan & Brown, 2017). As Wu et al. (2021) puts it, learners’ performance should be considered in a social context that was forgotten in the traditional assessment. In the same vein, Koné (2021) believed that learners’ progress should be evaluated by their cooperation. PBA, as opposed to the traditional assessment (TA), concentrates not only on the product of learning but also on the process of learning.

Reading comprehension refers to the process that involved various meaning-making procedures in inferring the implied meaning of a text (Rezai, Namaziandost, Miri, & Kumar, 2022, Rezai, Namaziandost, & Rahimi, 2022; Paris, 2005). The techniques used to teach reading are closely related to the employed techniques to evaluate reading comprehension (Nation, 2009). Evidence can be found in the literature that traditional standardized objective achievement tests focusing on multiple choices, matching, and true/false items are considered unsuitable and invalid to evaluate learners’ academic competencies (Azizi et al, 2022; O'Malley & Valdez Pierce, 1996; Michael, 1993; Nation, 2009). The traditional reading comprehension tests emphasized lower-order discrete, nonintegrated decoding skills, discrete point grammar, and decontextualized vocabulary items, which cannot provide a valid and reliable picture of the actual competencies of the learners (Jamali Kivi et al, 2021; Namaziandost et al, 2022). To compensate for the shortcomings of traditional reading comprehension tests, PBA was substituted in the educational context.

Aside the form of assessment, phycological factors may also influence the assessment process and its final results in language learning (Cao, 2022; Fong, 2022; Howard et al, 2021; Khajavy et al, 2021). Among psychological factors, AM shines differently because it has a supportive and enhancing role in the learners’ academic functioning and success (Caldarella et al, 2021; Martin et al., 2017; Peng, 2021). Generally, motivation is considered a stimulation force that shapes individuals’ behavior (Brophy, 1983). In the realm of education, student motivation, typically known as AM, is associated with their involvement in the process of learning (Hiver & Al-Hoorie, 2020). AM inspire learners to “make certain academic decisions, participate in classroom activities, and persist in pursuing the demanding process of learning” (Dörnyei & Ushioda, 2009, p. 2). That is, student AM pertains to students’ initial drive for launching and continuing the prolonged and tedious learning process (Ushioda, 2008). In the realm of language learning, Peng (2021) defined language learners’ AM as the extent to which they strive to learn a new language and their engagement in the process of learning.

Among student-related constructs, anxiety is one of the most experienced unpleasant emotions among learners (Burić & Frenzel, 2019; Shafiee Rad & Jafarpour, 2022), particularly among foreign language learners (Khajavy et al, 2018; Khajavy et al, 2021). Anxiety is a subjective state of fear and apprehension, which triggers rapid heart rate, hyperventilation, and sweating (Eysenck, 1992). According to Oteir and Al-Otaibi (2019), individuals feel anxious when they experience powerlessness in the face of an expected danger. In this regard, language anxiety is defined as situation-specific anxiety, including self-perceptions, beliefs, feelings, and behaviors experienced in the language learning classrooms (Horwitz et al, 1986; Liu et al, 2021; Sutarto Dwi Hastuti et al, 2022).

S-E is another student-attributed construct, which refers to “an individual's belief in his or her capacity to execute behaviors necessary to produce specific performance attainments” (Bandura, 1982, p.122). Simply put, S-E reflects confidence in the ability to exert control over one’s own motivation, behavior, and social environment. Previous studies reflected that S-E is associated with positive psychological constructs such as L2 grit (Zheng et al, 2022; Shabani et al, 2022), students’ academic achievement (Ma, 2022; Vadivel et al., 2022), critical thinking (Li et al, 2022), and inter and intra-relationships (Martin & Mulvihill, 2019; Vadivel & Beena, 2019). Academic S-E refers to “one’s confidence in his ability to successfully perform pro-academic self-regulatory behaviors- the degree to which students metacognitively, motivationally, and behaviorally regulate their learning process” (Gore, 2006, p.92). Learners’ S-E is influential in learners’ choice of tasks and their involvement in completing them (Olivier et al., 2018). According to Lai and Hwang (2016), efficacious learners show positive attitudes toward learning, and they relate their unsuccessful achievement to lower attempts rather than lower ability.

Due to the potent role of assessment in the realm of successful education, it is worthwhile to study the factors leading to its effective implementation. Yet, there is a dearth of literature about effective and convention assessment, particularly in EFL context. Therefore, more research is needed to fill this gap. PBA is one of the constructive assessments that can contribute to learners’ academic achievement. Despite the crucial role of PBA on RCA, AM, FLA, and S-E beliefs, only a few empirical studies have conducted in this regard, and to the best of our knowledge, no study has ever tried to investigate these variables simultaneously in EFL context. Thereby, it is less known about how PBA could facilitate language learning and inhibit learners’ motivation and S-E, as well as decrease FLA. Having this stand point in mind, the present study aims to explore the impact of performance-based assessment on RCA, AM, FLA, and S-E beliefs in the Iranian EFL context. Thus, the following research questions were put forth in the current study:

- RQ1: Does PBA have any significant effect on EFL learners’ RCA?
- RQ2: Does PBA have any significant effect on EFL learners’ AM?
- RQ3: Does PBA have any significant effect on EFL learners’ FLA?
- RQ4: Does PBA have any significant effect on EFL learners’ their S-E beliefs?

Based on the research questions, the following null hypotheses could be formulated:

- H01: PBA does not have any significant effect on EFL learners’ RCA.
- H02: PBA does not have any significant effect on EFL learners’ AM.
- H03: PBA does not have any significant effect on EFL learners’ FLA.
- H04: PBA does not have any significant effect on EFL learners’ their S-E beliefs.

## Review of the related literature

### The performance-based assessment (PBA)

The origin of TA is the behaviorist assumption that macro skills should be evaluated individually and sequentially (Salma & Prastikawati, 2021). In this regard, closed questions with one possible answer were applied to assess students’ progress. PBA is supported by the social-constructivist theory, which pinpointed that assessment is intervened with all the procedures involved in teaching/learning. The social-constructivist theory viewed that assessment should be designed in authentic tasks with student self-assessment and feedback (Shepard, 2000; Yan, 2021). The other related theoretical foundation for PBA goes back to Vygotsky’ s sociocultural theory describing learning as a social process and emphasizing on the role of social interaction and socially mediated communication in enhancing the learning process (Lightbrown & Spada, 2006; Wang, 2009).

Over the years, TA prevails in measuring the students’ skills with too much emphasis on the learning results to the point that they negate the actual learning competence (Alderson et al, 2017). Typically, TA involves multiple-choice tests, fill in the blanks, true-false, matching, short-answer types, and recall information. On the other hand, the advantages of performance-based assessment reside in its potential and perspectives to generate significant effects on the learning procedure where the active participation of the learners is emphasized (Koné, 2021). That is, performance assessment concentrates on observation and evaluation of the learners’ progress in action and on action (Fetsco & McClure, 2005).

In Koné’ s words (Koné, 2021), PBA asks the learners to activate their knowledge and skills from various domains to accomplish the required processes of a task. Taking a similar path, Griffith and Lim (2012) refer to PBA as an engaging tool to encourage the learners to apply their previous knowledge and abilities to solve a task. PBA is designed to put the learners in a situation to practice higher-order thinking skills, analyzing, and synthesizing (Herrera et al, 2013). Furthermore, PBA potentially informs how the learners authentically mastered the materials (Salma & Prastikawati, 2021) and provided meaningful information about the learners’ actual competencies (Gallardo, 2020).

### Academic motivation (AM)

AM is a critical element in a student’s psychological well-being and influence the way they behave. This concept refers to learners’ desire to academic subjects, which formulate their behavior, attitudes toward learning, and their attempts in the face of difficulties (Abdollahi et al, 2022; Koyuncuoğlu, 2021). Brophy (1983) characterized student AM into state motivation and trait motivation. State motivation captures learners’ tendency toward a special subject (Guilloteaux & Dörnyei, 2008). Trait motivation refers to learners’ general attitude toward the learning process (Csizér & Dörnyei, 2005). Based on Trad et al. (2014), trait motivation is static, whereas state motivation is dynamic and may change. Different factors may influence static motivation such as learning atmosphere and course content (Hiver & Al-Hoorie, 2020) as well as teachers’ personalities and relationships with the learners (Dörnyei, 2020; Kolganov et al, 2022).

Self-determination theory (SDT) introduced by Deci and Ryan (1985) was is the prominent theory in explaining AM. SDT proposed three components of motivation: (1) intrinsic motivation, (2) extrinsic motivation, and (3) amotivation. Extrinsic motivation touches upon the activities involved to reach a reward or avoid a punishment. In this regard, Deci and Ryan (2020) considered introjected regulation, identified regulation, and integrated regulation as three kinds of extrinsic motivation. Intrinsic motivation was generated from innate inspiration and inherent satisfaction to follow an activity. A motivation on the other hand refers to the condition of lacking the motivation to acquire an activity or to be involved in the learning process (Deci & Ryan, 2000; Vadivel et al., 2021). Both intrinsic and extrinsic motivations of students are influential in their AM (Al-Hoorie et al, 2022; Froiland & Oros, 2014). When pupils are highly motivated, they do not stop but enthusiastically follow the learning procedures (Martin, 2013). AM even preserves learners in the face of hardships that learners may encounter on the road of learning (Howard et al, 2021). Situated expectancy value theory (SEVT) is another theory that concentrated on AM (Eccles & Wigfield, 2020). This theory considers contextual determinants of students’ motivational perceptions, such as socializing agents and environmental/cultural influences, which form AM.

A plethora of recent studies highlighted the significant role of teachers’ positive communication skills such as closeness (e.g., Liu, 2021; Zheng, 2021), personality traits (Khalilzadeh & Khodi, 2018), confirmation (Shen & Croucher, 2018), and style of teaching (Dom’enech-Betoret & G’omez-Artiga, 2014) in improving AM among the learners. Additionally, Peng (2021) found out EFL teachers’ communication behaviors could foster learners’ AM and engagement. In another study, Koné (2021) confirmed that learners’ motivational and emotional states were significant in the success of performance-based assessment project. In the same line of inquiry, Arias et al (2022) examined the relationship between emotional intelligence and AM and their contributions to learners’ well-being. Their findings supported a strong correlation between these two variables. From another perspective, Cao (2022) discussed the mediating roles of AM and L2 enjoyment in learners’ willingness to communicate in the second language.

### Foreign language anxiety (FLA)

Rodríguez and Abreu (2003) define foreign language anxiety as situation-specific phenomenon caused by language learning in a formal situation, especially in a low self-appraisal of communicative competencies in that language. In the same vein, Horwitz et al (1986) proposed three components for foreign language anxiety (i.e., communication apprehension, test anxiety, and fear of negative evaluation). The first dimension, communication apprehension, refers to the anxiety in interacting with others, oral communication, or problems in listening comprehension. Test anxiety, the second component, is generated from a fear of failure in an examination. The third dimension, fear of negative evaluation, refers to apprehension about others’ evaluations and avoiding situations that may generate negative evaluation of others.

The attentional control theory (ACT) explains the reason why anxiety hinders learners’ academic achievement (Eysenck et al, 2007). ACT is originated from the processing efficiency theory (PET) by Eysenck and Calvo (1992) and explains that anxiety impairs attentional control by absorbing threat-related stimuli. Both external and internal causes may trigger students’ anxiety. Based on ACT, the high levels of worry and low self-confidence of anxious learners may be the reason of their unsuccessful performance (Eysenck et al, 2007). Different sources may evoke FLA. According to Alamer and Almulhim (2021), learners’ outlook on their language aptitude, their personality traits, the exposed experiences in the classroom, and the levels of difficulty may trigger anxiety. From another perspective, Young (1991) specified the student, the teacher, and the instructional practice as the potential sources of language anxiety. From Brown et al.’s viewpoint (Brown et al, 2001), students’ personality type (introversion vs. extroversion) can be among the possible causes of anxiety. As Cassady (2010a) stipulated, academic anxiety is a general term that refers to a groups of anxieties students experience in academic domain. According to him, students’ anxiety (e.g., test anxiety, math anxiety, foreign language anxiety, and science anxiety) interferes with students’ learning process. The focus of the present study is on FLA.

Leafing through the literature on FLA and its association with other student-related factors was quite rosy. For instance, the reciprocal relationships between foreign language anxiety and the following student-related factors were confirmed in the following studies: learners’ attitudes toward language acquisition (Horwitz et al, 1986), test performance (Cassady, 2010b; Covington et al, 1986), language performance (Zheng & Cheng, 2018), AM (Omidvar et al, 2013), anxiety, and social support as the predictor of AM during Covid-19 (Camacho et al, 2021). Furthermore, some studies in the realm of foreign/second language concentrate on the examination of skill-based anxiety and its role in language learning. For instance, speaking anxiety (Çağatay, 2015; Prentiss, 2021), listening anxiety (Zhang, 2013), reading anxiety (Hamada & Takaki, 2021a, 2021b), and writing anxiety (Zhang, 2019) were explored in recent years. These studies confirmed that FLA hinder language acquisition or performance. Via a structural equation modeling approach, Fathi et al (2021) documented that FLA and grit could predict learners’ WTC in EFL context. Similar findings were reached by Khajavy et al (2021). They came to conclusion that EFL learners’ FLA, WTC, and enjoyment were integrated. That is, WTC and enjoyment were positively correlated, but the relationship between FLA and WTC was significantly negative.

### Self-efficacy (S-E)

As Bandura (Bandura, 2012) defines, S-E impresses individuals’ understanding about their capabilities to execute appropriate behaviors for the purpose of achieving a desirable goal. S-E beliefs are influential in the ways people think, act, and used strategies in the face of different challenges (Bong & Clark, 1999). Social-cognitive theory espoused by Albert Bandura (1997) provides a theoretical foundation for perceptions of abilities that focus on the impact of self-referent phenomena and adopts an agentic view of personality. Bandura (1997) defined S-E as one’s perceived ability to effectively accomplish or demonstrate a behavior or series of behaviors in a given situation. Based on this agentic socio-cognitive perspective, the underlying features of personal agency include intentionality, forethought, self-reactiveness, and self-reflectiveness (Schunk & Mullen, 2012). As Bandura (2012) puts it, intention shapes future actions but for manifestation of forward-looking plans is more than an intentional state. Successful implementation of intentions and plans entails not only the intentional capability to make choices and action plans but also the capability to motivate and regulate the implementation of desired actions. This metacognitive capability is manifested through self-regulatory processes, which connect thought to action and include self-monitoring, performing self-guidance through personal standards, and corrective self-reactions (Cerit, 2019). Bandura’s agentic socio-cognitive theory (Bandura, 1997) also emphasized on individuals’ ability of reflecting on themselves, their thoughts, and actions.

Regarding students’ improvement in learning due to the role of S-E skills, different studies have been conducted. Enfield (2013) as well as Ha et al (2019) evinced the positive role of learners’ S-E in flipped classrooms. Furthermore, Doménech-Betoret et al (2017) concluded that expectancy-value beliefs affect academic S-E and the achievement/satisfaction relationship. The positive effects of student S-E beliefs and classroom engagement on their academic achievement were also concluded by the findings of Olivier et al (2018). In a recent attempt, Namaziandost and Çakmak (2020) inspected the difference that the flipped classroom made on S-E and gender. According to their findings, the females in the EG improved more than their male classmates when applying the flipped classroom activities. In the same line of inquiry, Yang et al (2022) concluded that learners’ L2 grit and S-E are correlated in the L2 context. They approved that teachers can set the tone of their learners’ S-E as well as L2 grit.

Taken together, the literature on the merits of PBA is rather flourishing; yet, there seems to be a lack of sufficient evidence on the extent and direction of its influence on boosting reading comprehension, motivation, foreign language learning anxiety, and learners’ S-E. Hence, the present study was conducted to fill this research gap in EFL context. The findings of this study can redound to the benefits of learners, teachers, policymakers, and curriculum designers.

## Methodology

The current study is quantitative in nature and uses a pretest-posttest quasi-experimental design. In the following, the undertaken steps are introduced in detail.

### Participants

Based on the results of the Oxford Quick Placement Test, 88 participants (45 females & 43 males) out of 154 freshmen EFL learners were chosen to take part in this research. These participants were at the intermediate level of English language proficiency. They were studying English teaching at the University of Gonabad, northeast of Iran. They were asked to not attend extra English classes this project. There were 45 students (24 females and 21 males) in the EG and 43 students (23 female and 20 male) in the CG with the age range from 18 to 23 and diverse socio-economic backgrounds. According to their syllabi, the students had to take a reading comprehension course in the first semester of the academic year (16 sessions). The students were fully aware of the voluntary nature of the study and gave informed consent to participate in this research project.

### Instruments

The following instruments were utilized in this research:

#### Oxford Quick Placement Test

The Oxford Quick Placement Test was employed to inspect the learners’ level of English language proficiency. In the Oxford Quick Placement Test, the range of score is from 0.1 to 0.9. The scores between 0.4 and 0.6 are considered as an intermediate level of English language proficiency. In the current study, the reliability of the Oxford Quick Placement Test was 0.89.

#### Achievement Motivation Scale (AMS)

To measure AM, the AMS college version (Vallerand et al., 1992) was utilized. This instrument was developed based on self-determination theory with 7 dimensions: intrinsic motivation toward knowledge, accomplishments, to undergo stimulation, external motivation as introjected, and identified regulations and amotivation. This scale consists of 28 items on a 7-point Likert scale (strongly agree to strongly disagree). The complete scores of this scale vary from 28 (the lowest range) to 196 (the highest range). As it was reported by Bahrani (2006), this scale presented satisfactory validity and reliability. In this study, the results of Cronbach’s alpha of 0.87 reflect significant reliability.

#### Foreign Language Anxiety Scale (FLCAS)

To probe into the level of learners’ anxiety in their foreign language classroom, the FLCAS developed by Horwitz et al (1986) was employed. This scale includes 33 items in a 5-point Likert scale (strongly agree to strongly disagree) to assess communication anxiety, fear of negative evaluation, test anxiety, and anxiety of foreign class. The reliability of the FLCAS estimated via Cronbach’s alpha (ranging from 0.81 to 0.77) was significant in this investigation.

#### The Self-Efficacy Scale (SES)

To gauge the degree of students’ beliefs about their successful achievement, the S-E Scale (Greene et al, 2004) was employed. This scale contains seven statements ranging from strongly disagree (1) to strongly agree (4). The reliability of this scale estimated through Cronbach’s alpha was acceptable (0.89).

### Procedures

A quasi-experimental design was used in the current research, and the participants were assigned to groups based on nonrandom criteria. To measure the students’ level of English language proficiency, the Oxford Quick Placement Test was utilized. The cut score (0.4–0.6) was considered (indicating intermediate level) to keep the learners in this project. Prior to administering the treatment, a pretest was administered to both groups. The pretest consisted of the designed reading comprehension test (which will be discussed in details in the following section), AMS, FLCAS, and SES.

After the pretest, the instruction was done by one of the researchers who was the instructor for both the experimental and CGs. To teach reading skills, Strategic Reading 2 (Richards & Eckstut-Didier, 2012) was utilized for both experimental and CGs. Students in the two groups of the study were exposed to these materials, with the exception that the students in the EG were exposed to PBA. In contrast, the CG ones were exposed to classroom traditional assessment. The major principle determining the focus of the teaching in PBA was to engage learners in all the learning activities. To do so, instruction involved working through the sequence of modeling, scaffolding, and guided practice in EG. Classes were considered as a source of learning as well as enjoyment. During their learning processes, EG was asked to evaluate their own learning progress. As the focus of this research was reading comprehension, regular instruction was administered to the students in the CG, and their books received no modification or supplementary parts. For the EG, in contrast, some modifications or supplementary sections were added to the books based on the underpinning theory of PBA. The reading tasks in EG were also modified in a way that learners had the opportunity to work in different peer or group activities.

To assess the reading skills of the students, the researchers of the present study designed a test to measure the learners’ reading comprehension skills before and after treatment. This assessment was designed based on Strategic Reading 2 (Richards & Eckstut-Didier, 2012) and included three sections to assess the knowledge of vocabulary, grammar, and the inferential reading comprehension skills. The first section, knowledge of vocabulary, involved 30 items. In the first ten items, the students were required to choose the word which was closest in meaning to the word phrase in bold given in the sentence. In the second group of items (10 items), some words were provided, and the students were asked to choose the equivalent definition from the four options. In the third subsection (10 items), a passage was provided, and students were required to fill in the blanks with appropriate words. All the vocabularies used in the assessment were selected from the reading passages in the students’ books. In the following section, a test of the grammatical knowledge consisted of 30 items in three parts. The first part (10 items) asked the students to put unscrambled words in order. In the second part (10 items), the students were asked to use appropriate forms of words in parentheses to complete the sentences. The third part (10 items) was designed to ask the students to check their knowledge and correct the errors. All tested grammatical structures were chosen from the students’ books. The last section in our designed assessment was inferential reading comprehension skills test, including three reading comprehension passages with 20 items. The questions asked the learners to infer the implied message of the passage, the tone of the text, the implicit possible relation between the two ideas in the text, and the conclusion of the text.

To inspect the face and content validity of the items, expert judgment was employed. In so doing, two psychometricians and two English teachers were invited to evaluate the quality of the items. Based on their comments, some items were revised. Following this step, the test was a sample of 34 university students similar to the target population to check the test-retest reliability. To gauge the stability of the results over time, the same test was readministered to the same participant after 2 months. Based on the results of the Pearson correlation coefficients, a high test-retest reliability of the test was confirmed (*r* = 0.90, *p* < 0.05).

Furthermore, in each reading session, the students in the EG were asked to complete a knowledge chart as well as a self-assessment checklist. In the knowledge chart, the students were required to complete a chart with three columns. The title of each column is as follows: what I know about the topic of the text, what I want to know, and what I learned from reading the text. The self-assessment checklist with three sections was designed to evaluate the pre-reading, while reading, and post-reading strategies of the learners. Students in the EG are asked to reflect on their employed reading strategies and assess their own progress. After completing the knowledge chart and self-assessment checklist, they were kept in a portfolio designed for each student. The teacher read the portfolio of each participant and gave feedback to them. The learners understood where they were in relation to their learning goals so that they could evaluate their progress, identify gaps or misconceptions in their understanding, and take remedial actions. Students were also provided with some insights into future-oriented solutions. Thinking aloud and writing journals, sharing experiences with their peers are among other strategies applied for the EG. The participants in the CG received no treatment. At the end of the experiment, after the instruction was completed, a posttest was run to investigate the effectiveness of the program in both the control and EGs. The posttest comprised of the designed reading comprehension test, AMS, FLCAS, and SES.

### Data analysis

To compare the pretest and posttest scores of the EG and CG learners with respect to RCA, AM, AA, and S-E, one-way MANOVA was utilized. This statistical test is used when there is one independent variable (in this case, the PBA, which surfaces as the experimental vs. control groups) and two or more related dependent variables (RCA, AM, AA, and S-E in this case). Prior to the administration of MANOVA, its assumptions (including normality, sample size, outliers, linearity, homogeneity of regression) were checked.

## Results

In this section, the results of the statistical analysis are displayed. Tables 1 and 2 deal with comparing the EG and CG learners on the speaking pretest.

**Table 1:** Descriptive statistics results comparing EG and CG on RCA, AM, FLA, and S-E scores of the pretest

	Groups	Mean	Std. deviation	*N*
RCA, pre	EG	11.9222	1.87373	45
	CG	11.7907	1.23072	43
	Total	11.8580	1.58468	88
AM, pre	EG	79.4000	18.24879	45
	CG	81.1628	26.85049	43
	Total	80.2614	22.74319	88
FLA, pre	EG	72.6667	10.96896	45
	CG	64.0000	20.75022	43
	Total	68.4318	16.96162	88
S-E, pre	EG	19.2444	1.90878	45
	CG	19.1628	1.86356	43
	Total	19.2045	1.87641	88

**Table 2:** MANOVA results comparing EG and CG on RCA pretest, AM pretest, FLA pretest, and SS-E pretest

Effect		Value	*F*	Hypothesis df	Error df	Sig.	Partial eta squared
Groups	Pillai’s trace	.079	1.774	4.000	83.000	0.142	.079
	Wilks’ lambda	0.921	1.774	4.000	83.000	0.142	.079
	Hotelling’s trace	.086	1.774	4.000	83.000	0.142	.079
	Roy’s largest root	.086	1.774	4.000	83.000	0.142	.079

The pretest mean scores of the EG and CG for RCA, AM, FLA, and S-E are shown in Table 1. There were minimal differences between the mean scores of the two groups on all RCA of speaking except for FLA (for which the mean scores of the EG were higher than CG). To make sure whether the differences were of statistical significance or not, the researcher had to refer to the MANOVA table below (Table 2).

Since the most commonly reported statistics is Wilk’s lambda, here the value for this statistic is reported (0.921). The Wilk’s lambda’s associated *Sig.* value was found to be 0.142, which is larger than the significance level (i.e., 0.142 > .05). This shows that the two groups of EG and CG were not significantly different on their pretest in terms of the dependent variables. What follows is the results of a similar data analysis procedure performed for the RCA, AM, FLA, and S-E posttest scores of the EG and CG. Any possible changes on the posttest could be attributed to the treatment provided for the EG (that is, using the PBA).

Based on Table 3, considering RCA, the score of participants in the EG is higher than their counterpart in the CG (*M* = 17.13; *SD* = 1.45). Regarding AM, EG got higher score in the posttest than CG (*M* = 103.31; *SD* = 18.70). Moreover, EG received higher score than CG in the posttest of FLA (*M* = 54.44; *SD* = 10.94). EG also got higher score than the CG in the posttest of S-E (*M* = 54.44; *SD* = 10.94). To find out whether these differences were statistically significant or not, the researcher needed to consult to the MANOVA table below (Table 4).

**Table 3:** Results of descriptive statistics for RCA posttest, AM posttest, FLA posttest, and SS-E posttest

Descriptive statistics				
	Groups	Mean	Std. deviation	*N*
RCA, post	EG	17.1333	1.45149	45
	CG	14.3372	1.57632	43
	Total	15.7670	2.05939	88
AM, post	EG	103.3111	18.70321	45
	CG	80.7442	22.43731	43
	Total	92.2841	23.42361	88
FLA, post	EG	54.4444	10.94107	45
	CG	107.5349	22.75475	43
	Total	80.3864	31.98255	88
S-E, post	EG	23.5111	2.19112	45
	CG	21.4419	1.85552	43
	Total	22.5000	2.27429	88

**Table 4:** MANOVA results comparing EG and CG on RCA posttest, AM posttest, FLA posttest, and SS-E posttest

Effect		Value	*F*	Hypothesis df	Error df	Sig.	Partial eta squared
Groups	Pillai’s trace	0.881	146.501	4.000	79.000	.000	0.881
	Wilks’ lambda	0.119	146.501	4.000	79.000	.000	0.881
	Hotelling’s trace	7.418	146.501	4.000	79.000	.000	0.881
	Ro’s largest root	7.418	146.501	4.000	79.000	.000	0.881

The Wilk’s lambda’s associated *Sig.* value was .00, which is lower than the significance level (.00 < .05). A *p*-value less than or equal to the significance level shows that there was a significant difference between the two groups. Thus, the two groups of EG and CG were significantly different on their posttest in terms of RCA, AM, FLA, and SS-E. Now to see which of the four dependent variables caused the difference between the two groups, Table 5 should be looked at.

**Table 5:** Results of tests of between-subject effects for RCA posttest, AM posttest, FLA posttest, and SS-E posttest

Source	Dependent variable	Type 3 sum of squares	df	Mean square	*F*	Sig.	Partial eta squared
Groups	RCA, post	145.153	1	145.153	228.590	.000	0.736
	AM, post	6874.898	1	6874.898	94.242	.000	0.668
	FLA, post	59077.854	1	59077.854	286.020	.000	0.777
	S-E, post	98.698	1	98.698	115.596	.000	0.690
Error	RCA, post	52.069	82	0.635			
	AM, post	5981.864	82	72.950			
	FLA, post	16937.235	82	206.552			
	S-E, post	70.013	82	0.854			
Total	RCA, post	22245.750	88				
	AM, post	797173.000	88				
	FLA, post	657644.000	88				
	S-E, post	45000.000	88				
Corrected total	RCA, post	368.974	87				
	AM, post	47733.898	87				
	FLA, post	88990.864	87				
	S-E, post	450.000	87				

Because we are looking at a number of separate analyses here, it is suggested that we use a more stringent significance level to avoid type 1 error. The most common way of this is to apply Bonferroni adjustment, which entails dividing the significance level (i.e., .05) by the number of analyses. In this case, since there were four dependent variables, significance level ought to be divided by four (giving a new significance level of .012). The results now are significant if the probability value (*Sig.*) is less than .012. In Table 4, reading through group row, under the *Sig.* column, the *p*-value for all the four dependent variables (.000) was found to be less than .012. All other *p*-values, however, were larger than the significance level. This means that RCA, AM, FLA, and S-E significantly differed in the EG and CG due to the treatment provided for the EG learners. More importantly, partial eta squares of 0.736, 0.668, 0.777, and 0.690 for RCA, AM, FLA, and S-E, respectively, are considered quite large effect sizes.

## Discussion

The present study was an attempt to explore the impacts of PBA on RCA, AM, FLA, and S-E among EFL learners. Based on data analysis, classroom performance-based assessment had a significant effect on the improvement of reading comprehension skills among EFL learners. In this way, the students in the EG outperformed their peers in the CG with regard to the usage of PBA to assess reading comprehension. Furthermore, the results revealed that PBA significantly influence the learners’ AM. In addition, a significant difference was found in AA and sense of efficacy among the control and EGs in favor of PBA.

Regarding the first research question (RQ: does PBA have any significant effect on EFL learners’ RCA?), the potential benefits of PBA implementation in the successful enhancement of reading comprehension skills in EFL learners were confirmed. This outcome can be attributed to the characteristics of PBT: the usage of authentic materials for the purpose of real-life communication, monitoring the learners’ development, providing deep insight into the degree and depth, engaging learners in the cooperative task, and activating self-aid skills (e.g., S-E, self-awareness, self-evaluation, and self-regulation) among learners. Theoretically, this finding is in line with the propositions of the socio-constructivist theory, which emphasizes on the crucial role of self-monitoring and awareness in the enhancement of the learning progress (Herrera et al, 2013). As was mentioned before, PBA opens a window for practicing thinking, problem-solving skills, self-evaluation, self-monitoring, and self-awareness among the learners. The depth of classroom performance-based assessment helps teachers capture learning goals and processes. Furthermore, the picture that classroom PBA provides from the students’ learning is much clearer than TA. With this clearer picture, decision-making about ongoing instruction and future activities is much easier and more accountable in the educational system.

PBA also revolves around Vygotsky’s sociocultural theory highlighting the idea that social interaction is central to learning. Cooperative activities and students’ social interaction are among the underpinning criteria for designing classroom PBA, which is attributed to the peculiar features of Vygotsky’s sociocultural theory. This situation motivates university learners to become autonomous learners. Creating and maintaining rapport as well as the use of interaction are among the significant objectives of PBA, which are supported by Vygotsky’s sociocultural theory. Providing nonthreatening situations and maintaining friendly communication work as affective glue and helps learners, in particular university students to perform better.

The current findings are also in accord with prior studies (e.g., Abualrob & Al-Saadi, 2019; Narathakoon et al, 2020; Sumardi, 2017; Wiyaka & Prastikawati, 2019) on the general efficacy of classroom PBA. Strong reading comprehension is the ultimate goal of reading instruction at all grade levels, but research on the realm of PBA is still in its infancy and calls for more identical investigation. In their study, Suastra and Menggo (2020) concluded that PBA empowers learners to write skillfully. In PBA, university professors are actively involved in their learning processes as well as their evaluation. They are asked to reflect on their weaknesses, and problem-solving activities in line with their needs are suggested, which enhances efficient learning. From another perspective, Efendi (2017) confirmed that the implementation of PBA for writing skills helps both English teachers as well as learners. The applicability of PBA in the improvement of speaking skills was supported by the findings of Soto et al. (2018). In their study, some performance-based tasks were introduced to boost the learners’ speaking skills. Furthermore, the contribution of PBA in enhancing listening comprehension among EFL learners was concluded in an investigation by (Abd El Ghany et al, 2019).

Regarding the second finding of the study (RQ2: does PBA have any significant effect on EFL learners’ AM?), the significant influence of PBA on the learners’ AM is defined by the objectives of PBA as mentioned earlier and self-determination theory. It means that when university students’ competence, satisfaction, and autonomy are fulfilled, they become self-determined and motivated. The main objectives of PBA suggest that it can foster a positive self-image and offer support and encouragement, which motivates university students to act enthusiastically. This finding is also congruent with some previous studies which evidence the profound influence of PBA in boosting learners’ writing skills and motivation (Menggo et al., 2019), a more friendly learning atmosphere, and thus increased S-E attributes and positive feelings of the learners (AlKhateeb, 2021), self-confidence and motivation (Soto et al., 2018), and positive emotional state and motivation. Through PBA, university students are informed about their progress; they can set new goals for their learning. According to Dörnyei et al (2016) as well as Al-Hoorie et al (2022), L2 learning is guaranteed when the learner is determined and motivated to achieve their goals.

The other findings of the present research indicated that due to the positive effects of PBA, students in the EG were able to manage their FLA much better than their counterparts in the CG. Thus, the third null hypothesis is rejected (PBA does not have any significant effect on EFL learners’ FLA). It can be inferred that fostering university students’ involvement in their learning process and asking them to evaluate themselves while learning give them a sense of self-worth and self-confidence, thus decreasing their anxiety. This outcome is also supported by the underpinning theories of ACT (Eysenck et al, 2007). Based on ACT, FLA can be an obstacle against students’ progress. According to Djafri and Wimbarti (2018), different factors may induce language anxiety, such as teaching style, teacher-student relationship, assessment, and final results. In this regard, Aydin et al (2006) pointed out that test anxiety considerably affects learners’ language skills and achievement; therefore, they recommended that training courses for teachers and examiners to envision their outlook on the influence of test anxiety on the learning process. Put it another way, Aydin et al (2020) stated that students with test anxiety lose their concentration and do not perform well on the test. More significantly, language performance and test anxiety are at opposite poles (Khoshhal, 2021); language performance will increase if test anxiety decreases. In such a situation, that text anxiety hinders the language learning process; PBA can assist language learners in relieving their stress and anxiety.

The effectiveness of using PBA in enhancing learners’ S-E was also confirmed by the results of this study. Thereby, the fourth null hypothesis (PBA does not have any significant effect on EFL learners’ their S-E beliefs) was rejected. As Suryadi and Santoso (2017) maintained, S-E is an important predictor of their achievement. More importantly, S-E is a complex construct affecting AM and learning process (Yasemin & Cavus, 2014). Thus, it can be inferred that PBA puts the university students at the center of the learning processes and assumes assessment as an option to envision their S-E, self-awareness, and self-evaluation; experiencing such a situation increases the probability of successful achievement. The suggested tasks of PBA could enable learners to improve their weaknesses during the processes of learning without anxiety; this opportunity consequently directs learners toward a positive viewpoint. Efficacies of learners can skillfully tackle the difficulties in the path of language learning. This finding is in accord with those of Villarta et al (2021), which concluded that PBA could foster learners’ S-E in science classes. In the same line of inquiry, AlKhateeb (2018) provided evidence that PBA supported leaners’ achievement and S-E. Yan et al (2022) also conducted a study with the aim of providing instructional intervention in self-assessment and approved that this intervention could boost learners’ S-E in creativity and academic performance.

## Conclusion and implications

All in all, the findings of the current study indicated that PBA has a beneficial effect on RCA, AM, FLA, and S-E among EFL learners. The outcome calls for more attention to the role of PBA in the L2 learning process. Due to the fact that teaching and assessment influence every aspect of L2 learning process, their significant role in L2 classroom contexts should be acknowledged and valued by all of the stakeholders in the field, including policymakers, curriculum designers, materials developers, and teachers. As the findings of this study reflected, PBA could foster RCA.

This rational can be put forward that the helpful strategies involved in PBA activate language learners to be involved in reading comprehension activities. Thus, implementing PBA is strongly recommended in language classes with the aim of helping students learn and practice RCA as well as speaking, listening, and writing more effectively. In so doing, the crucial role of teachers in applying PBA in the L2 classrooms should be highlighted. They need to acquire the related knowledge about the advantages of PBA and how to implement PBA in their classes. Teachers also need to learn which strategies are useful and convenient for practicing PBA in enhancing all four main skills. Adding the implications of the current study in pre-service and in-service teacher training programs can pave the way for providing language teachers with the skills and knowledge required to implement PBA.

It was also concluded that learners’ psychological and cognitive associated factors such as AM, foreign language learning anxiety, S-E, and consequently their academic achievement could be affected by the employed assessment, especially in higher education. Therefore, critical inspection is necessary to evaluate and modify the way students in general and university students in particular are taught and assessed to guarantee the well-being of education. Further attention is also needed in pre-service and in-service teacher and university professor training programs to learn about the importance of learners’ psychological and cognitive attributed constructs and useful strategies to improve them.

In the present research, the learners’ years of teaching experience, educational levels, age, and gender were not considered. It is suggested to consider these variables in similar research studies in future. The possible effect of different learners’ sociocultural backgrounds was not under focused in the current study. Future study may address the influence of PBA on EFL learners’ skills with respect to different sociocultural backgrounds. Future research can investigate the effect of PBA on other areas of L2, such as listening, speaking, writing, and pronunciation. Furthermore, this study was quasi-experimental design; future research can apply other methods to ensure the generalizability of the findings. Future studies can also conduct a similar study with more participants. Additionally, it should be noted that in the current research, only reading comprehension was considered, and the other three main skills were not the target of this study. Thus, considering the influence of PBA on speaking, listening, and writing skills of the learners can be considered as future research perspectives. Exploring the influence of PBA on other learner-related constructs such as self-regulation, L2 grit, academic buoyancy, and coping strategies is suggested for future studies.

## Data Availability

The authors declare that the data supporting the findings of this study are available within the article.

## Abbreviations

PBA: Performance-based assessment
L2: Second/foreign language
RCA: Reading comprehension achievement
AM: Academic motivation
FLA: Foreign language anxiety
SS-E: Students’ self-efficacy
EG: Experimental group
CG: Control group
TA: Traditional assessment
SDT: Self-determination theory
SEVT: Situated expectancy value theory
ACT: Attentional control theory
PET: Processing efficiency theory
OQPT: Oxford Quick Placement Test
AMS: Achievement motivation scale
FLCAS: Foreign Language Anxiety Scale
SES: The self-efficacy scale
RQ: Research question
